# The use of complementary and alternative medicine products in preceding two days among Finnish parents - a population survey

**DOI:** 10.1186/1472-6882-11-107

**Published:** 2011-11-04

**Authors:** Katri P Hämeen-Anttila, Ulla R Niskala, Sanna M Siponen, Riitta S Ahonen

**Affiliations:** 1 Finnish Medicines Agency, Kuopio, Finland (P.O. Box 55, Microkatu 1, FI-00301 Helsinki, Finland; 2 School of Pharmacy, Faculty of Health Sciences, University of Eastern Finland, P.O. Box 1627, 70211 Kuopio, Finland

## Abstract

**Background:**

The use of complementary and alternative medicines (CAM) has been extensively studied globally among adult and paediatric populations. Parents, as a group, had not been studied to assess their knowledge and attitude to CAM and general medicine use. This study is necessary since parents' attitude to medicine use is known to influence their child's attitude to medicine use later in life. We therefore aim to assess the extent and types of CAM use among Finnish parents, and to determine the factors that promote the CAM use. Also, we aim to determine parents' attitude to general medicine use.

**Methods:**

Children less than 12 years old, as of spring 2007, were identified from the database of the Finnish Population Register Centre and were selected by random sampling. The parents of these children were identified and a questionnaire was sent to them. Only the parent who regularly takes care of the child's medicine was requested to fill the questionnaire. Cross-tabulations and Chi-square test were used to determine the associations between categorical variables. CAMs were defined as natural products that are not registered as medicines, such as homeopathic preparations, dietary food supplements, and traditional medicinal products.

**Results:**

The response rate of the survey was 67% (n = 4032). The use of CAM was 31% in the preceding two days. The most commonly used CAM products were vitamins and minerals, followed by fish oils and fatty acids. Prescription and OTC medicines were used concomitantly with CAM by one-third of the parents. CAM was frequently used by parents over 30 years (33%), female parents (32%), highly educated parents (35%), and parents with high monthly net income (3000-3999 euros, 34%). The users of CAM had more negative attitudes towards medicines than non-users of CAM.

**Conclusions:**

Our findings are in accordance with those of previous studies that women over 30 years of age with a high education and income typically use CAMs. Finnish parents seem to use CAMs as complementary rather than alternative to medicines. Health care professionals should take into consideration both the concomitant use as well as the negative attitudes among CAM users in encounters with the parents.

## Background

The use of complementary and alternative medicines (CAM) has been widely studied among adult populations. In Europe, the prevalence of the use of CAM among adults, including both products and therapies, has varied from 6-49% according to different studies with varying recall periods [1-6]. However, parents as a group, had not been studied to assess their knowledge and attitude to CAM, and general medicine or CAM use. Parental CAM use and attitudes are important since these may influence their own health behaviours and how they medicate CAM and other medicines to their children. The influence of parents, especially the mothers, on health related orientations and on children's expectations to take medicines are known to be strong [7,8].

Little is known about how attitudes relate to use of CAMs. There is evidence that those who have negative attitudes toward medicines tend to prefer natural treatments [9]. Furthermore, those who have concerns about taking long-term medications and fear of side-effects of medicines may tend towards using CAMs [10]. It is generally perceived that CAM use is harmless [11]. However, more studies are needed to confirm these findings.

CAM use is more prevalent among women [3,12-14], middle-aged between 30 and 65 years [1,3,5,13], and people who are highly educated [3,15,16]. In addition, the use of CAM seems to be more common in high income class than in low income class [2,13]. Moreover, the use of CAM has been found to be less common in low social classes than in high social classes [17].

Other factors have been shown to predict the use of CAM. The use of CAM seems to be associated with visits to a physician and the use of conventional medicine [3,6,16]. Furthermore, smoking has been found to decrease the use of CAM [6]. Chronic diseases and poor self-reported health status are known to be associated with higher CAM use [3,15,16].

Earlier studies were focused on adult population or limited to a certain populations, such as cancer patients or pain patients [14,18-21]. Studies are lacking on parental use of CAM and their association with attitudes towards general medicine use. The aim of this study is to describe the prevalence of the use of CAM among parents of under 12-year-old children, and the factors associated with it, including attitudes toward medicines among the users and non-users of CAM.

## Methods

### Definitions

The National Centre for Complementary and Alternative Medicine (NCCAM) defines CAM relatively broad as methods, practices and products that have no place in conventional medicine [22]. We used the NCCAM definition as a basis for our definition, however, in this study, treatments considered as CAM are excluded. We defined CAM as natural products that are not registered as medicines in Finland, such as homeopathic preparations, dietary food supplements, and traditional medicinal products. Thus, OTC-medicines and prescription medicines are not included as CAMs in our study.

In Finland, there is no congruent legislation for CAM. CAM is not generally covered either by Social Insurance Institution or by private insurances in Finland [23]. CAMs may be bought from grocery shops, health food shops, and also from pharmacies [24]. On the other hand, medicines, both over-the-counter (OTC) and prescription, can only be bought from pharmacies. The Social Insurance Institution reimburses the patient for a part of the cost of prescribed medicines

### Setting

Finland is a country with approximately 5 million inhabitants. The health care has been organized on three levels: primary health care is offered by the district health centers. District hospitals offer secondary care and five university teaching hospitals tertiary care, i.e, most advanced medical care in the country. Responsibility for organizing the health care services is at the municipalities (local government).

In Finland, health care is financed mainly by taxes, and primary health care is available to all citizens for ambulatory medical services in health centers, free of charge for children and for a small fee for adults. Outpatient visits can also be made to private health care clinics.

### Data collection and participant selection

This cross-sectional population survey was conducted in Finland in the spring of 2007 (February-April). The survey was conducted in order to explore children's medicine use and health status [25]. Thus, a simple random sample (n = 6000) of children under 12 years of age was taken, instead of a random sample of parents. Parental CAM use and attitudes toward medicine use were explored, since children's medicine and CAM use at this age is still controlled by the parents and their attitudes are influencing on actual medicine and CAM use by children.

The database of the Finnish Population Register Centre was used to randomise children under 12 years. This database contains constantly updated information on everyone living permanently in Finland. The parents of these children were identified and a postal questionnaire was sent to the home address of the parents of each child, preferably the mothers. It was requested on the questionnaire that the key person who takes charge of the child's medication should fill the questionnaire. The child's name was printed on the questionnaire in order to specify the child in the families with two or more children. Two reminders were sent in order to increase the response rate.

### Questionnaire desing

The questionnaire consisted of 30 questions, both structured and open-ended. It included questions about the use of CAM products and medicines by the parent; the parent's attitudes toward medicines; child's health; child's use of CAMs and medicines; sources of information about medicines; and demographic characteristics of responding parent and the child. It was designed to be comparable with the questionnaires used in the previous studies carried out in Finland [26,27]. The questionnaire was first piloted with a convenient sample of 61 mothers, whose answers were not included in the main study. Minor modifications were made on the basis of the pilot-test.

The main outcome measure was parental CAM use in preceding two days in order to limit recall bias. A classification of reported CAM use was made by the research group for following groups: vitamins and minerals, probiotics, fish oils and fatty acids, homeopathics, and other CAMs (including, e.g., preparations for cold; ginger preparations; preparations for warts, muscle pain, dieting, stomach function, and constipation; aloe vera products; other ointments). Parents' attitudes to medicines were measured with 21 Likert items, which were created by the researchers based on literature and validated [28]. Half of these items were general in nature (e.g., *Medicines are necessary in treating illnesses*), and the other half were connected to the medicine used by the child (e.g., *Side effects of children's medicines worry me*).

Guidelines by the Finnish National Advisory Board on Research Ethics http://www.tenk.fi/en/index.html were followed in carrying out the study. The data management and disposal of all personal data were conducted in accordance with national privacy protection laws.

### Data analysis

Data were analyzed with SPSS for Windows statistical software, Release 14.0 (SPSS Inc., Chicago, IL, USA). The categorical variables were cross-tabulated and their potential dependencies were estimated with *x*^2 ^tests. *P *values less than 0.05 were considered as significant.

## Results

A response rate of 67% (n = 4032) was obtained. The final study sample was representative in age and gender of children less than 12 years old living in Finland [24]. The regional distribution of the children differed slightly from that of the target population: Southern region, 40.7% (the actual proportion of the children in that area is 41.1%); Western, 32.6% (34.9%); Eastern, 12.8% (9.9%); Oulu region, 10.1% (10.4%); Lapland, 3.4% (3.3%); and Åland 0.5% (0.5%). On the other hand, the analysis of the non-respondents showed no differences from the target population (i.e., children under 12 years) in age, gender, or regional distribution.

The majority of those who completed the questionnaire were mothers (95%). Table 1 shows that many of respondents were either working or studying (67%) and about a quarter of them were at home looking after the children. The median age was 36 years (range 18-61). Of the parents, 31% (n = 1242) had used some CAM in the preceding two days. Moreover, the use of prescribed (p < 0.001) or over-the-counter (p = 0.027) medicines predicted the use of CAM. Of the CAM users, 36% was concomitantly using prescribed medicines and 34% over-the-counter medicines (Table 1). There was a significant difference in the age, gender, use of prescribed and OTC medicines, levels of education, and monthly net income of the parents who were CAM users and CAM non-users (Table 1). However, their working status did not differ significantly.

**Table 1:** Characteristics of the study population and CAM use by these characteristics of the respondents

Characteristic	No. (% of the responses, n = 4032)	CAM use (% of the respondents, n = 1242)	CAM non users (% of the respondents, n = 2720)	Pearson *x*^2 ^*P*
Age, years				p < 0.001
Under 30	686 (17.2)	165 (24.4)	510 (75.6)	
30-39	2126 (53.4)	684 (32.5)	1418 (67.5)	
40 and over	1172 (29.4)	380 (33.0)	772 (67.0)	
Gender				p < 0.001
Male	177 (94.8)	26 (14.9)	148 (85.1)	
Female	3830 (4.4)	1205 (32.1)	2552 (67.9)	
Prescribed medicine use				p < 0.001
No prescribed medicine use	2409 (60.2)	680 (29)	1706 (71.5)	
One or more prescribed medicines	1591 (39.8)	558 (36)	1007 (64.3)	
OTC medicine use				p = 0.027
No OTC medicine use	3078 (77.5)	927 (30.4)	2120 (69.6)	
One or more OTC medicines (vitamins not included)	893 (22.5)	302 (34.4)	577 (65.6)	
Education				p < 0.001
Junior high school or less (≤ 9 years)	252 (6.3)	54 (22.0)	191 (78.0)	
Senior high school/vocational school (11-13 years)	2456 (61.4)	740 (30.6)	1679 (69.4)	
Polytechnic, college or university degree (≥ 15 years)	1291 (32.3)	441 (34.5)	839 (65.5)	
Working status				p = 0.788
Working or studying	2698 (67.3)	826 (31.0)	1835 (69.0)	
Home with children	1119 (27.9)	355 (32.2)	748 (67.8)	
Not working (including persons on sick leave, retired, and unemployed)	194 (4.8)	60 (31.3)	132 (68.8)	
Monthly net income of the household in euros				p = 0.009
≤ 1999 €	797 (20.7)	214 (27.3)	569 (72.7)	
€ 2000-2999	1292 (33.5)	401 (31.5)	874 (68.5)	
€ 3000-3999	1419 (36.8)	481 (34.3)	923 (65.7)	
€ 4000-10000	347 (9.0)	103 (30.3)	237 (69.7)	

Altogether, 1818 different CAM products were reportedly used by the parents who were CAM users. On average, parents were using 1.5 different CAM products (range 1-9 products). The most commonly mentioned CAMs were vitamins and minerals followed by fish oils and fatty acids (Table 2). Homeopathics were least mentioned products. There were statistical differences in the use of different CAM products between different groups of parents. For example, there was a statistically significant difference in the use of fish oils and fatty acids between different aged parents, and furthermore, in the use of vitamins and minerals between parents with different educational background (Table 2). Other differences may be seen in Table 2.

**Table 2:** CAM use by selected characteristics (n = 4032)

Characteristic	Vitamins and minerals n (%)	Fish oils and fatty acids n (%)	Probiotics n (%)	Homeopathics n (%)	Other* n (%)
Age, years	p = 0.136	p < 0.001	p = 0.504	p = 0.584	p < 0.004
Under 30 (n = 686)	110 (16.0)	42 (6.1)	13 (1.9)	3 (0.4)	32 (4.7)
30-39 (n = 2126)	406 (19.1)	253 (11.9)	57 (2.7)	16 (0.8)	149 (7.0)
40 and over (n = 1172)	202 (17.2)	160 (13.7)	28 (2.4)	10 (0.9)	103 (8.8)
Gender	p < 0.001	p = 0.178	p = 0.084	p = 0.646	p = 0.001
Male (n = 177)	12 (6.8)	12 (6.8)	0	0	7 (4.0)
Female (n = 3808)	703 (18.5)	445 (11.7)	98 (2.6)	30 (0.8)	279 (7.3)
Prescribed medicine use	p = 0.219	p < 0.001	p < 0.001	p = 0.065	p < 0.001
No prescribed medicine use (n = 2409)	419 (17.4)	236 (9.8)	41 (1.7)	23 (1.0)	143 (5.9)
One or more prescribed medicines (n = 1591)	301 (18.9)	221 (13.9)	58 (3.6)	7 (0.7)	145 (9.1)
OTC medicine use	p = 0.22	p = 0.266	p = 0.183	p = 0.743	p = 0.007
No OTC medicine use (n = 3078)	531 (17.3)	362 (11.8)	83 (2.7)	24 (0.8)	202 (6.6)
One or more OTC medicines (vitamins not included) (n = 893)	184 (20.6)	93 (10.4)	17 (1.9)	6 (0.7)	82 (9.2)
Education	p < 0.001	p = 0.172	p = 0.079	p = 0.019	p = 0.074
Junior high school or less (≤ 9 years) (n = 252)	35 (13.9)	20 (7.9)	1 (0.4)	0	9 (3.6)
Senior high school/vocational school (11-13 years) (n = 2456)	403 (16.4)	291 (11.8)	61 (2.5)	13 (0.5)	179 (7.3)
Polytechnic, college or university degree (≥ 15 years) n = 1291)	281 (21.8)	145 (11.2)	36 (2.8)	16 (1.2)	97 (7.5)
Working status	p < 0.001	p = 0.006	p = 0.175	p = 0.052	p = 0.057
Working or studying (n = 2698)	440 (16.3)	328 (12.2)	68 (2.5)	14 (0.5)	199 (7.4)
Home with children (n = 1119)	251 (22.4)	101 (9.0)	31 (2.8)	14 (1.3)	69 (6.2)
Not working (including persons on sick leave, retired, and unemployed) (n = 194)	31 (16.0)	29 (14.9)	1 (0.5)	2 (1.0)	21 (10.8)
Monthly net income of the household in euros	p = 0.003	p = 0.534	p = 0.261	p = 0.062	p = 0.918
≤ 1999 € (n = 797)	116 (14.6)	84 (10.5)	12 (1.5)	7 (0.9)	54 (6.8)
€ 2000-2999 (n = 1292)	223 (17.3)	144 (11.1)	34 (2.6)	15 (1.2)	98 (7.6)
€ 3000-3999 (n = 1419)	294 (20.7)	177 (12.5)	40 (2.8)	5 (0.4)	105 (7.4)
€ 4000-10000 (n = 347)	63 (18.2)	40 (11.5)	8 (2.3)	1 (0.3)	25 (7.2)

*Includes, e.g., preparations for cold; ginger preparations; preparations for warts, muscle pain, dieting, stomach function, and constipation; aloe vera products; other ointments

In 10 of the 21 statements assessing the attitude towards medicines, a statistically significant difference between the users and non-users of CAM were found (Table 3). The users of CAM considered medicines more negative than the non-users of CAM. This was seen both in general statements about medicines and statements related to a child's medicine use. Based on the responses to the statements, parental attitudes may influence in their management of their children's health concerns. For example, the users of CAMs responded statistically significantly more often that they try to avoid giving medicines to their child (69%, p < 0.001), and instead, try to take care of their child's ailments by some other means than using medicines (37%, p = 0.003) compared to the parents of CAM non-users (63% and 32%, respectively) (Table 3).

**Table 3:** The proportion users and non-users of CAM who agree* with the statements (n = 4032) (significant p-values in bold)

Statement	CAM user n = 1242% (n/all CAM users)	Non-users of CAM n = 2725% (n/all non-users of CAM)	Pearson *x*^2 ^*P*
**General statements**			
Medicines are necessary in treating illnesses.	88 (1088/1236)	88 (2372/2720)	0.470
Prescription medicines are effective.	75 (932/1236)	76 (2072/2723)	0.639
Interactions of medicines worry me.	68 (837/1236)	60 (1635/2722)	**< 0.001**
Prescription medicines are safe.	65 (799/1236)	69 (1872/2723)	**0.011**
Over-the-counter (OTC) medicines are safe.	55 (679/1236)	56 (1524/2723)	0.545
The more you need to use analgesics the less effective they are for pain.	54 (672/1235)	56 (1532/2722)	0.273
OTC medicines are effective.	45 (555/1236)	43 (1167/2722)	0.233
Medicines can disturb the body's own capability to heal illnesses.	43 (533/1236)	39 (1051/2720)	**0.008**
Medicines are unnatural to the human body.	27 (328/1236)	21 (567/2721)	**< 0.001**
Long-term use of analgesics reduces the pain threshold.	25 (312/1236)	26 (697/2722)	0.808
Medicines are dangerous, even when used according to the instructions.	8 (92/1234)	5 (135/2723)	**0.002**
**Statements connected to child's medicine use**			
Medicines that a doctor has prescribed for the child are necessary.	77 (955/1236)	81 (2193/2722)	**0.017**
Side-effects of children's medicines worry me.	69 (854/1236)	61 (1663/2721)	**< 0.001**
I try to avoid giving medicines to my child.	69 (848/1236)	63 (1709/2723)	**< 0.001**
I take care of my child's minor ailments by using OTC medicines	69 (848/1236)	66 (1798/2721)	0.117
I take my child to see a doctor only when other ways of treatment do not help.	49 (609/1236)	48 (1294/2723)	0.307
Doctors prescribe antibiotics to children too easily.	48 (589/1236)	43 (1157/2721)	**0.003**
Fewer, a natural means of defense of the child's body, should not be lowered artificially with medicines.	37 (460/1236)	35 (945/2723)	0.126
I try to take care of my child's ailments by some other means than using medicines.	37 (452/1236)	32 (866/2722)	**0.003**
I usually give less analgesic to the child than is recommended in the instructions.	24 (294/1236)	25 (687/2722)	0.327
The child needs to learn how to bear the pain.	10 (125/1236)	9 (235/2724)	0.132

*agree & totally agree

## Discussion

The use of CAM among parents was found to be common. Third of the parents had used some CAM products in the preceding two days. The most commonly used CAM products were vitamins and minerals followed by fish oils and fatty acids. Only few mentioned that they had used homeopathics. Our study supports earlier findings which suggest that female gender [12-14,29], age group of approximately 30-45 years [1,3,5,13] as well as high education and high income is associated with the CAM use [2,3,13,15,16].

As found in the previous studies, the users of CAM in our study had generally more negative attitudes towards medicines than non-users of CAM [9-11]. However, also the concomitant use of prescribed medicines or over-the-counter medicines and CAM was common in our study. In any way, health care professionals should take into account the use of CAMs by their patients and realize that the users often have a cautious attitude towards using medicines, but on the other hand, also concomitant use of CAMs and medicines do occur. Safe and rational use of both medicines and CAMs is especially important when the patient is a child. By openly discussing the attitudes of the parent, a treatment plan with both CAMs and medicines may be safely agreed for the child.

Some limitations of the study need to be pointed out. The study sample was taken from the children under 12 years, not the parents. The sample is representative in age and gender of children under 12 years. Most parents in this study were relatively young (30-45 years old) due to the fact that they were parents of children under 12 years. It is most likely that majority of them did not have serious medical conditions which would warrant chronic use of either conventional medicines or CAMs. Furthermore, 95% of the respondents were mothers and only 4% were fathers. Therefore, this study reflects more the use of CAMs among Finnish mothers than those of Finnish parents in general. Furthermore, statistical comparison between the genders may not be valid. The use of CAMs was asked from previous two days, which decreases the possibility of recall bias. However, it is possible that this time window skewed the data towards CAMs used for daily health maintenance and patterns of CAM use over time may not have been captured that well. Future studies are needed to explore the use of CAM therapies among Finnish parents, and furthermore, if the parental attitudes have an influence in their management of their children's health concerns.

## Conclusions

Our findings are in accordance with those of previous studies that women over 30 years of age with a high education and income typically use CAMs. Finnish parents seem to use CAMs as complementary rather than alternative to medicines. Even so, the CAM users have a more negative attitude towards medicines than the non-users.

## Competing interests

The authors declare that they have no competing interests.

## Authors' contributions

UN performed the statistical analysis for the study. KH-A drafted the manuscript, and with SMS and RSA, she participated in the design of the study and acquisition of the data. Furthermore, UN, SMS and RSA revised the manuscript critically. All authors have read and approved the final manuscript.

## Pre-publication history

The pre-publication history for this paper can be accessed here:

http://www.biomedcentral.com/1472-6882/11/107/prepub
